# Consumption of Nutritionally Enriched Hen Eggs Enhances Endothelium-Dependent Vasodilation via Cyclooxygenase Metabolites in Healthy Young People—A Randomized Study

**DOI:** 10.3390/nu15071599

**Published:** 2023-03-25

**Authors:** Petar Šušnjara, Zrinka Mihaljević, Ana Stupin, Nikolina Kolobarić, Anita Matić, Ivana Jukić, Zlata Kralik, Gordana Kralik, Anđelina Miloloža, Tihana Pavošević, Vatroslav Šerić, Zdenko Lončarić, Darko Kerovec, Olivera Galović, Ines Drenjančević

**Affiliations:** 1 Institute and Department of Physiology and Immunology, Faculty of Medicine, Josip Juraj Strossmayer University of Osijek, J. Huttlera 4, HR-31000 Osijek, Croatia; psusnjara@mefos.hr (P.Š.); zrinka.mihaljevic@mefos.hr (Z.M.); ana.stupin@mefos.hr (A.S.); nikolina.bilic.dujmusic@gmail.com (N.K.); anitaa3006@gmail.com (A.M.); ivana.jukic@mefos.hr (I.J.); 2 Scientific Center of Excellence for Personalized Health Care, Josip Juraj Strossmayer University of Osijek, Trg Svetog Trojstva 3, HR-31000 Osijek, Croatia; zkralik@fazos.hr (Z.K.); gkralik@fazos.hr (G.K.); ogalovic@kemija.unios.hr (O.G.); 3 Department of Animal Production and Biotechnology, Faculty of Agrobiotechnical Sciences, Josip Juraj Strossmayer University of Osijek, Vladimira Preloga 1, HR-31000 Osijek, Croatia; 4 Department of Clinical Laboratory Diagnostics, Osijek University Hospital, J. Huttlera 4, HR-31000 Osijek, Croatia; andjelina.m@gmail.com (A.M.); tihanapavos@gmail.com (T.P.); vatroslav.seric@mefos.hr (V.Š.); 5 Department for Agroecology and Environment Protection, Faculty of Agrobiotechnical Sciences, Josip Juraj Strossmayer University of Osijek, Vladimira Preloga 1, HR-31000 Osijek, Croatia; zdenko.loncaric@fazos.hr (Z.L.); darko.kerovec@fazos.hr (D.K.); 6 Department of Chemistry, Josip Juraj Strossmayer University of Osijek, Ul. Cara Hadrijana 8a, HR-31000 Osijek, Croatia

**Keywords:** microcirculation, endothelium, vasodilation, cyclooxygenases, functional food, eggs, reactive hyperemia

## Abstract

Objective: The present study aimed to evaluate the effects of enriched hen egg consumption on endothelium-dependent vasodilation (EDV) and the role of cyclooxygenases in EDV in the microcirculation of young healthy individuals. This study hypothesizes that Nutri4 eggs will improve endothelial function, which will be manifested by changes in microcirculatory flow measured by a laser Doppler flowmeter (LDF) during reactive hyperemia in response to vascular occlusion, in which *n*-3 PUFA plays an important role as well as its degradation pathway by cyclooxygenases. Materials and Methods: Participants consumed three eggs per day for three weeks: The control group (CTRL, *n* = 14) consumed regular hen eggs (approximately 0.330 mg of lutein, 1.785 mg of vitamin E, 0.054 mg of selenium and 438 mg of *n*-3 PUFAs daily) and Nutri4 group (*n* = 20) consumed enriched eggs (approximately 1.85 mg of lutein, 0.06 mg of selenium, 3.29 mg of vitamin E, and 1026 mg of *n*-3 PUFAs daily). Skin microvascular blood flow in response to EDV (post-occlusive reactive hyperemia (PORH) and iontophoresis of acetylcholine (AChID)) and sodium nitroprusside (SNPID; endothelium-independent) was assessed by laser Doppler flowmetry before and after dietary protocol and in a separate group of participants who were administered perorally 100 mg of indomethacin before microvascular response assessment. Arterial blood pressure, heart rate, serum lipid, and liver enzymes, anthropometric measurements, protein expression of cyclooxygenase 1 (COX-1), cyclooxygenase 2 (COX-2), neuronal nitric oxide synthases (nNOS), inducible nitric oxide synthases (iNOS), and endothelial nitric oxide synthases (eNOS) were measured before and after dietary protocol. Results: PORH and AChID were significantly enhanced, and SNPID remained unchanged in the Nutri4 group, while none was changed in the CTRL following a respective diet. PORH decreased after administration of indomethacin in Nutri4 after dietary protocol. Protein expression of COX-2 was significantly higher in the Nutri4 group compared to the CTRL after the dietary protocol. Conclusion: Consumption of enriched eggs improves microvascular EDV in healthy young subjects. Results suggest an element of *n*-3 PUFAs metabolites via the cyclooxygenases pathway in enhanced reactive hyperemia.

## 1. Introduction

The consumption of *n*-3 polyunsaturated fatty acids (*n*-3 PUFAs), (i.e., eicosapentaenoic acid (EPA), docosahexaenoic acid (DHA), and alpha-linolenic acid (ALA)) has been recognized to reduce risks for cardiovascular (CV) diseases [1]. *n*-3 PUFAs demonstrated protective CV effects by lowering blood pressure (BP) [2] and exhibiting anti-inflammatory, anti-atherosclerotic, and anti-oxidant properties [3]. For example, the addition of EPA and DHA to daily diet was shown to decrease the concentration of serum lipids, most notably triglycerides, in individuals with hyperlipidemia [4]. Interestingly, additional beneficial effects of *n*-3 PUFA consumption contributed to the alteration of vascular tone and blood vessel reactivity [5], both in healthy people and in CV patients [1,2,5]. Adding fatty acids, such as fish oil, to a daily diet induced increased vascular reactivity in healthy patients [4]. Endothelium-dependent vasodilation (EDV) is the process of widening blood vessels that occurs when the endothelium, a thin layer of cells that lines the interior of blood vessels, relaxes [5]. This process plays a crucial role in regulating blood flow and maintaining blood pressure in the body and tissue perfusion [6]. Measuring EDV is important because it can provide insight into the health of blood vessels and endothelial function [7,8]. Endothelial dysfunction, which can manifest as reduced EDV, is associated with various diseases including atherosclerosis, hypertension, diabetes, and cardiovascular disease [9]. Furthermore, the increased proportion of EPA and DHA in the diet increases vasodilator mechanisms in the microcirculation of the forearm, which can contribute to lowering blood pressure [5,10]. In various clinical trials, it was shown that *n*-3 PUFAs have a cardioprotective effect by improving arterial hemodynamics and reducing arterial stiffness, which can positively interfere with the process of atherosclerosis [6,7]. Cyclooxygenases (COX) are enzymes that play a key role in the conversion of arachidonic acid, an *n*-6 polyunsaturated fatty acid, into various bioactive metabolites, including prostaglandins and thromboxanes. These metabolites are involved in many physiological processes such as inflammation, pain, and fever vasoreactivity to different stimuli [5].

There are two isoforms of COX enzymes, COX-1 and COX-2. COX-1 is constitutively expressed in most tissues and is involved in maintaining normal physiological functions, while COX-2 is mainly induced in response to pro-inflammatory stimuli and is involved in inflammation and pain [8].

The COX pathway has been extensively studied due to its potential role in the pathogenesis of various diseases, including cardiovascular disease. Nonsteroidal anti-inflammatory drugs (NSAIDs) are known to inhibit COX enzymes, leading to a reduction in the production of prostaglandins and thromboxanes, which can result in the relief of pain and inflammation and altered vasoreactivity. However, the inhibition of COX enzymes may also lead to unwanted side effects, such as gastrointestinal bleeding, renal dysfunction, and increased cardiovascular risk [8]. Interestingly, COX metabolites of EPA are involved in *n*-3 PUFA-mediated vasodilation [8]. Evidence is accumulating that the consumption of *n*-3 PUFAs in a form of functional foods (e.g., *n*-3 PUFA-enriched hen eggs) may improve microvascular endothelium-dependent vasodilation in healthy young individuals and in patients with metabolic diseases [5,6,7]. Generally, the investigation of the effects of PUFAs has used doses ranging from 1 to 4 g per day, but the optimal dose can depend on the specific health condition being treated [6,9].

In addition to *n*-3 PUFAs, other nutritional elements may play an important role in preserving vascular ones with anti-oxidant properties. Vitamin E, which is the most abundant anti-oxidant soluble in fats, has been described to alleviate hypertension, diabetes mellitus, fatty liver disease, etc. [11,12,13]. In cells exposed to hypoxia, vitamin E had a protective effect by reducing ROS and cell apoptosis [8,14], while it may also prevent functional vascular impairment, as observed in diabetic rats [15]. Another very important essential trace element that has a cardioprotective role in human health is selenium (Se) [16]. Se is a major component of glutathione peroxidase, an anti-oxidant enzyme that catalyzes the reduction of hydrogen peroxide to water and oxygen, as well as the reduction of peroxide radicals to alcohol and oxygen, which protects cells from oxidative damage [17]. Se deficiency itself has been reported in patients with stroke, atherosclerosis, and diabetes [18], as well as in hypertension and atherothrombotic diseases [19]. Lutein is also associated with a decrease in blood pressure due to increased NO production, a decrease in atherosclerotic lesions, and a decrease in monocyte chemotaxis [20]. Consumption of lutein reduced the risk of metabolic syndrome, and exhibited protection in the progression of early atherosclerosis [21], which was attributed to its anti-oxidant and anti-inflammatory effects [20]. However, there is a lack of studies in animal models or humans that evaluate the health effects of these nutrients the components of functional food. Although we are aware of the controversial role of egg consumption on serum lipid levels, in our previous studies [6,7,8,9] we have shown that consumption of eggs in this protocol has not increased serum lipid concentrations in studies’ participants. Similarly, other groups have not shown an increase in serum lipid levels after the consumption of enriched eggs [7,8]. Interestingly, in our recent study by Ćurić et al. [9] the consumption of *n*-3 PUFA-enriched hen eggs even reduced LDL cholesterol in the serum of cardiovascular patients. 

Therefore, the present study hypothesized that a diet containing hen eggs enriched with *n*-3 PUFAs, vitamin E, lutein, and selenium would improve endothelial function manifested by changes in microcirculatory blood flow. The present study aimed to determine the effect of consumption of these enriched hen eggs on microvascular reactivity in response to endothelium-dependent and endothelium-independent stimuli in young healthy subjects of both sexes and to identify potential mechanisms involved in altered vascular reactivity.

## 2. Materials and Methods

### 2.1. The Study Population

Thirty-four young healthy individuals participated in this study. Eligibility criteria included age range between 18 and 30 years, arterial BP values ≤ 120/80 mmHg, and normal body mass index (BMI; 18.5–24.9 kg/m^2^). Exclusion criteria were a history of smoking, hyperlipidemia, renal impairment, hypertension, coronary artery disease, diabetes, cerebrovascular and peripheral artery disease, and taking any drugs or substances that could affect the endothelium and they were assessed during the first study visit using a verbal questionnaire and a questionnaire of eating habits of the participants declaring none of the participants have been taking enriched functional food or *n*-3 PUFAs, lutein, selenium and vitamin E supplementation in any form prior or during the enrollment in the present study. Written informed consent was obtained from each subject. 

### 2.2. Study Protocol

**Figure 1:**
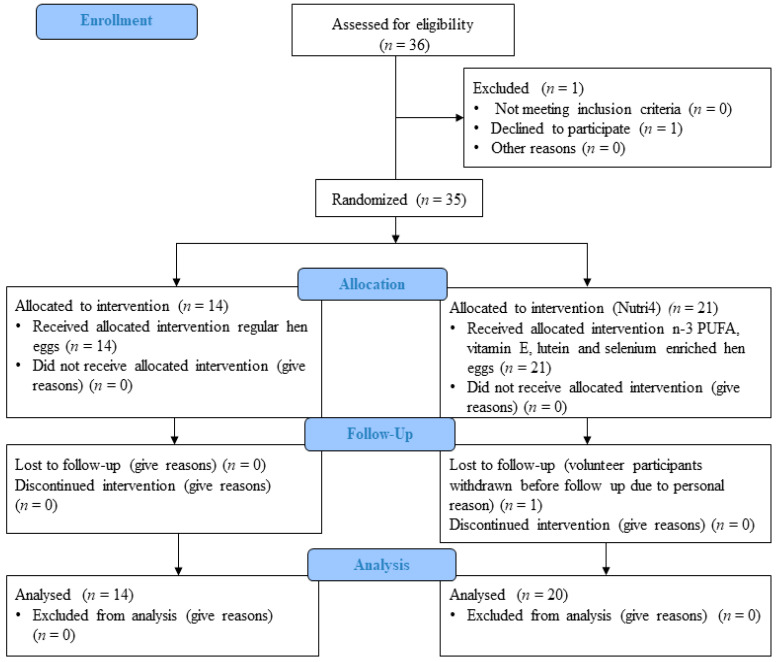
Consort study protocol.

The study protocol and procedures conformed to the standards set by the latest revision of the Declaration of Helsinki and were approved by the Ethical Committee of the Science Center of Excellence, Josip Juraj Strossmayer University of Osijek (Cl: 602-04/14-08/06; No: 2158-610714-114) and Ethics Committee of the Medical Faculty Osijek (Cl: 602-04/20-08/07, Registration number: 2158-61-07-20147). The study was registered on the clinical trial under the title: Effect of Enriched QUARTET^®^ Hen Eggs (Marijančanka d.o.o. Marijanci, Croatia) on Cardiovascular Function in Cardiovascular Patients and Healthy Individuals. NCT number: NCT04564690 [22]. Figure 1 represents the CONSORT flow chart of the study. This was a randomized, double-blinded, prospective, placebo-controlled interventional study. The study protocol lasted 21 days. During those three weeks’ subjects were instructed to eat three hen eggs per day (a total of 63 eggs). Subjects were divided into two study groups: a control group, consuming regular eggs (CTRL, *n* = 14), and a group consuming enriched eggs (Nutri4, *n* = 20). Nutri4 consumed approximately 3.29 mg/per day of vitamin E, 1.85 mg per day of lutein, 0.06 mg/per day of selenium and 1026 mg/per day of *n*-3 PUFAs in three eggs per day for 3 weeks), while CTRL consumed regular hen eggs produced on the same farm (approximately 1.79 mg/per day of vitamin E, 0.33 mg/per day of lutein, 0.05 mg/per day of selenium and, 438 mg/per day of *n*-3 PUFAs) in three eggs per day for 3 weeks.

The study was conducted at the Faculty of Medicine in Osijek, and the participants were young healthy medical students who were recruited during both the winter and summer semester in 2020. Participants were recruited and included using a survey questionnaire to meet the inclusion and exclusion criteria of the study, then were given the code ZCI-Q-1-No. or ZCI-Q-2-No., in which ZCI-Q- denotes the name of the project under study (“Scientific Center of Excellence” in which hen eggs enriched with four nutrients are tested: *n*-3 PUFA, vitamin E, selenium and lutein. Number 1 or 2 denoted group (CTRL or Nutri4, depending on the eggs to be consumed), and were assigned by the person who divided the eggs. No. is the ordinal number of subjects (1, 2, 3…). 

After recruitment, a simple randomization procedure was performed by drawing A or B for each subject, i.e., in which group, A (CTRL) or B (Nutri4). The result of such randomization is the difference in the number of subjects that exists between the CTRL and Nutri4, but the number of subjects in both groups met the sample size required for adequate statistical analysis [23]. 

All eggs were the same L size and produced on the poultry farm Marijančanka d.o.o. Marijanci, Croatia. Enriched hen eggs were produced according to the protocol of the research group from the Faculty of Agrobiotechnical Sciences Osijek, Josip Juraj Strossmayer University of Osijek, in which rapeseed oil (1.5%) in feed mixtures fed to laying hens were replaced with mixture of fish (1.5%), linseed (2%) oil and 0.43 selenium mg/kg mixture, 100 mg/kg mixture of vitamin E and lutein. The content of eggs is shown in Table 1, in which Kralik et al. performed an analysis on uncooked eggs [24,25].

Subjects were instructed to boil the eggs (10 min) before consumption every morning during the three-week protocol. Additionally, all subjects were instructed to take only the eggs given to them for the study (total of 63 eggs) and not to take the other food rich in *n*-3 PUFAs, vitamin E, lutein and selenium, any other enriched functional food or any other supplementation during the study protocol. The study was performed in the Laboratory for Clinical and Sport Physiology, Department of Physiology and Immunology at the Faculty of Medicine, University of Osijek. Two study visits and all the measurements described below were done on the first day and the day immediately after the end of the dietary protocol. All measurements and blood sampling occurred in the morning after overnight fasting. Subjects were instructed not to undertake any strenuous activity during the 24 h preceding the visit and to avoid caffeine intake in the morning before the study visit.

### 2.3. Anthropometric and Arterial Blood Pressure Measurements 

The subject’s weight (kg) and height (m) were measured to calculate body mass index (BMI). Waist and hip circumference, heart rate (HR), and arterial blood pressure (BP) were measured at the beginning of each visit after 15 min rest in the seated position using an automated oscillometric sphygmomanometer (OMRON M3, OMRON Healthcare Inc., Osaka, Japan). The final values of BP and HR were the means of three repeated measurements.

### 2.4. Assessment of Microvascular Endothelium-Dependent and Endothelium-Independent Vasodilation

Measurement of microvascular blood flow and reactivity tests were performed in all subjects using laser Doppler flowmetry (LDF) (MoorVMS-LDF, Axminster, UK), before and after corresponding diet protocols, as previously described [7,9]. Endothelium-dependent vasodilation of skin microcirculation was tested by measurement of microvascular response to vascular occlusion (post-occlusive reactive hyperemia, PORH) and to iontophoresis of acetylcholine (acetylcholine-induced dilation, AChID), while endothelium-independent vasodilation was assessed by iontophoresis of sodium nitroprusside (sodium nitroprusside-induced dilation, SNPID). All microvascular tests were performed according to the protocols described in detail in earlier publications of our research [7,8,26,27] group. LDF measurements were performed in the morning after overnight fasting, in a temperature-controlled room (23.5 °C +/− 0.5 °C). Microvascular blood flow was expressed in arbitrary perfusion units (PU) and described as the area under the curve (AUC) using the original software provided by the manufacturer (MoorVMS-PC v4.0, Axminster, UK). PORH measurement was expressed as the difference between the percentage of flow change during reperfusion and occlusion in relation to baseline (R-O%). AChID and SNPID were expressed as flow increases following ACh or SNP administration compared to baseline flow. 

In addition, to test the role of metabolites of cyclooxygenase enzymes (COX-1 and COX-2) in the microvascular responses, in a separate set of experiments, measurements of ACh-induced dilation and PORH were conducted in randomly selected subjects prior and 90 min after peroral intake of 100 mg of indomethacin (nonselective inhibitor of the COX-1 and COX-2 enzymes) [28] to examine the influence of metabolites of the cyclooxygenase pathway of *n*-3 PUFA degradation on the endothelial function of young healthy subjects at both study visits (*n* = 6 from CTRL and *n* = 5 from Nutri4 group).

### 2.5. Isolation Peripheral Blood Mononuclear Cell (PBMC)

Venous blood was sampled in vacutainers with the anticoagulant EDTA. The venous blood was then mixed with 1 × phosphate-buffered saline (PBS) and layered on a Ficoll-Paque^®^ PLUS solution (density gradient) and centrifuged for 25 min at 800× *g* (Rotina 380, Hettich GmbH & Co. KG, Tuttlingen, Germany). After centrifugation, the middle layer) of PBMCs were separated and used in further experiments [29]. 

### 2.6. Protein Expression of Enzymes Important in the Mechanisms of Microvascular Reactivity

To determine the relative protein expression of iNOS, eNOS, nNOS, COX-1 and COX-2 from isolated PBMC, Western blot was carried out according to the well-established method at our laboratory [30,31,32]. Homogenization buffer (50 mM HEPES 150 mM NaCl, 1 mM EDTA, 10% glycerol, 1% TRIXON-X), protease inhibitor cocktail 0.4 µL/100 µL (Complete mini EDTA free Protease inhibitor cocktail, Roche Diagnostics GmbH, Mannheim, Germany) was used to homogenize PBMC. A total of 200 µL of homogenization buffer was added to resuspend 1 × 107 PBMC. Everything is well shaken on a mixer (vortex) and kept on ice for 3 min. This was followed by centrifugation of the samples for 30 min at 15,000× *g* at 4 °C. The supernatant was pipetted to be used for further analysis. Sample preparation for SDS-PAGE electrophoresis followed. Samples were boiled at a ratio of 1:1 at 95 °C for 5 min with Laemmli buffer, centrifuged briefly (spin-down) and applied to the gel. After electrophoresis, the samples were transferred to a PVDF membrane and then incubated with the primary antibody overnight (COX-1 rabbit PolyAB, Proteintech Europe, Manchester, UK, #13393-1-AP, 1:500; COX-2 rabbit PolyAB, Proteintech Europe, UK, #12375-1-AP, 1:500; iNOS rabbit PolyAB, Novus Biological, Engelwood, CO, USA, #NB300-605, 1:500; eNOS rabbit PolyAB, Novus Biological, USA, #NB300-500, 1:1000; nNOS rabbit PolyAB, Novus Biological, USA, # NBP1-39681, 1:1000) overnight at 4 °C. The next day, the membrane was incubated with appropriate horse radish peroxidase (HRP)-labeled secondary antibody (goat anti-rabbit HRP, Abcam, Cambridge, UK, ab205718; goat anti-mouse HRP, Santa Cruz Biotechnology, Dallas, TX, USA, sc-2005, both in 1:7500 dilution), followed by chemiluminescence imaging using Pierce ECL Western Blotting Substrate (Thermo Scientific, Rockford, IL, USA) according to the manufacturer’s instructions. Protein expression levels were normalized to the expression of β-actin and presented as relative protein levels. 

### 2.7. Venous Blood Sample Analysis

Venous blood samples were taken after 15 min resting in a seated position at each visit. Blood samples were analyzed for full blood count, fasting blood glucose, creatinine, urea, fasting lipid profile (total cholesterol, high-density lipoprotein (HDL) cholesterol, low-density lipoprotein (LDL) cholesterol, and triglycerides), and liver enzymes determined by spectrophotometric method, hsCRP and transferrin determined by immunoturbidimetry, plasma electrolytes determined by potentiometry methods. These analyses were performed at the Department of Clinical Laboratory Diagnostics, University Hospital Osijek.

### 2.8. Measurements of Serum Fatty Acids Profile

The serum fatty acid profile was determined by gas chromatography–tandem mass spectrometry (GC–MS/MS), a system by Thermo Fisher GC Trace 1300 coupled with a TSQ 9000 Triple Quadrupole. For the preparation of standard solutions, a solution of fatty acid methyl esters (FAME MIX) was purchased as 30 mg/mL of the total concentration of fatty acids in methylene chloride from Supelco (Supelco Inc., Bellefonte, PA, USA). Before analysis, samples were stored at −80 °C and prepared for analysis according to Wang et al. [33]. Shortly, to 200 μL of serum sample in an Eppendorf tube, an additional 200 μL of saline, 1 mL of methanol and 2 mL of chloroform was added to and mixed for 10 min on a rotary stirrer. Then 500 μL of 1M NaCl was added and stirred for another 2 min. After stirring, the sample was centrifuged for 10 min at 3000 rpm at room temperature. After centrifugation, the layers were separated and 1 mL of the lower chloroform layer was taken into a glass tube and evaporated to dryness under a stream of nitrogen. Fatty acid methylation was started by adding 1 mL of 14% methanolic BF 3 to the evaporated sample. The sample is then placed on a 75 °C thermoblock for 45 min. After cooling, 1 mL of highly purified water and 1 mL of hexane are added and stirred for 2 min. After separating the layers, 600 μL of the upper hexane layer was evaporated to dryness. The sample was recovered with 600 μL of hexane and analyzed The separation was performed on a capillary column 30 m in length, with an internal diameter of 0.25 mm, and film thickness of 0.25 μm (DB-225MS; Agilent Technologies, Santa Clara, CA, USA). A 1 μL aliquot of prepared sample solution was injected into the GC split injector (split ratio 1:15) for GC analysis, using the following conditions: helium (6.0) as a carrier gas with the constant flow of 1.2 mL/min, oven temperature profile initially 60 °C for 2 min, then ramped to 130 °C at 8 °C/min, held for 2 min, then ramped to 220 °C at 5 °C/min, held for 15 min and finally raised to 230 °C at a rate of 10 °C/min and held for 5 min. The inlet temperature was maintained at 230 °C, the MS transfer line temperature was maintained at 260 °C and the ion source temperature at 250 °C. The total run time was 53 min. Serum fatty acids profile analysis was performed at the BIOCentre’s Bioanalytical Laboratory, BIOCentre—incubation center for biosciences, Zagreb, Croatia [6]. 

### 2.9. Measurements of Serum Vitamin E Concentration 

Vitamin E concentrations in serum samples were determined according to the existing protocol [34]. First, absolute ethanol was used to denature serum proteins and Xylene was used to separate the supernatant from proteins. The whole mixture was centrifuged at 3000× *g* for 10 min and the supernatant was separated. 2,2-bipyridyl and FeCl_3_ were then added to the supernatant, resulting in pink coloration. After 2 min of incubation, the absorbance was measured using a spectrophotometer (PR 3100 TSC Microplate Reader, Bio-Rad Laboratories, Hercules, CA, USA) at 492 nm, and the obtained absorbance was proportional to the serum vitamin E concentration. A five-point calibration curve was also made.

### 2.10. Serum Selenium Concentration Measurements

All samples for measurement of serum Se concentration were digested in ultra-pure HNO_3_ and H_2_O_2_ (5:1 ratio) at 180 °C for 60 min in a close microwave system CEM Mars 6 (CEM, Matthews, NC, USA). Se concentrations in solutions of digested serum samples were determined by inductively coupled plasma mass spectrometry (ICP-MS) (ICP-MS, Agilent 7500a, Agilent Technologies Inc., CA, USA). Each serum sample on the ICP was analyzed by internal pooled plasma control and reference material NIST 1567b (wheat flour, National Institute of Standards and Technology, Gaithersburg, MD, USA) was used for the control of the analytical method. All samples were analyzed in triplicate. Measurements were performed at the Department for Agroecology and Environment Protection, Faculty of the Agrobiotechnical Sciences Josip Juraj Strossmayer University of Osijek.

### 2.11. Measurements of Serum Lutein Concentration

Lutein concentrations in serum samples were determined according to the existing protocols [35,36]. In 200 μL of serum, we added 1 mL of deionized water and 0.01% ascorbic acid dissolved in absolute ethanol and stirred the mixture. Then we added 2 mL of hexane, stirred and centrifuged it at 2500 rpm for 20 min. After centrifugation, we separated the supernatant, evaporated the supernatant, and determined the concentration of lutein using HPLC. HPLC analysis was performed at the Department of Chemistry, Josip Juraj Strossmayer University of Osijek.

### 2.12. Statistical Analysis

The results are presented as arithmetic mean and standard deviation (SD). The Shapiro–Wilkinson test was used to assess the normality of data distribution. The differences in the normally distributed numerical variables between the two independent groups were tested by the Student’s *t*-test, in the case of deviation from the normal distribution by the Mann–Whitney U-test. A paired *t*-test was used for intragroup comparisons or Wilcoxon rank sum tests when variables were not normally distributed. The One-Way ANOVA test was used for comparison between multiple groups. The level of statistical significance was determined at *p* < 0.05. 

The statistical program SigmaPlot (version 11.2, Systat Software, Inc., Chicago, IL, USA) was used. The sample size required to show a potentially significant effect was calculated based on preliminary data from the same research group where the primary outcome was an LDF measurement collected from eight subjects [6]. To detect differences in primary outcomes recorded in this study (PORH measurements) with a significance level of 0.05 and a statistical power of 80% for the paired *t*-test, the required sample size was 13 subjects per group. For the group with indomethacin primary outcomes recorded in this group are PORH measurements after intake of indomethacin which is five subjects per group.

## 3. Results

### 3.1. Effects of Diets on Anthropometric and Biochemical Measurements in Study Population

Characteristics of the study population are presented in Table 2. There was no difference in participants’ age between the CTRL and Nutri4 groups. Subjects of both sexes from the Nutri4 and CTRL groups were lean and had similar BMI and WHR before the study protocol. No significant change in BMI or WHR in the Nutri4 group and CTRL group occurred after the dietary protocol. There was no difference in BP (SBP, DBP, and MAP, respectively) between study groups before and after the dietary protocol. The Nutri4 group had significantly higher HR than the CTRL group before the dietary protocol and after the three-week dietary protocol (Table 2).

Before the dietary protocol, all biochemical parameters were similar between groups. Consumption of eggs did not cause significant changes in serum concentration of HDL cholesterol and triglycerides in CTRL and Nutri4 group compared to pre-diet measurements. However, the serum LDL cholesterol concentration was significantly increased in the CTRL group after a three-week dietary protocol. The iron level in the Nutri4 group decreased significantly after dietary protocol (although within the reference interval), There was a significant increase in serum glucose and urea concentration in Nutri4 subjects after egg consumption compared to the CTRL group (however, within the laboratory reference range for population, which is used as a parameter for diagnostic purposes of an individual’s health). Other measured parameters were similar between groups and not affected by respective dietary protocols. (Table 2).

### 3.2. Free Fatty Acids Profile, Vitamin E, Lutein, and Selenium in Serum

The concentration of EPA and vitamin E was significantly increased in the Nutri4 group following enriched egg consumption compared to the initial value and compared to the values of the CTRL group. DHA concentration was significantly increased in Nutri4 groups following enriched egg consumption and in the CTRL group after egg consumption. The serum concentration of lutein was significantly increased in the Nutri4 group after the dietary protocol, while in the CTRL group, there was no statistical change after the dietary protocol. Selenium concentration remained statistically unchanged after the three-week dietary protocol in the CTRL group and the Nutri4 group (Table 3). 

### 3.3. Skin Microvascular Reactivity to PORH, Acetylcholine, and Sodium Nitroprusside 

**Figure 2:**
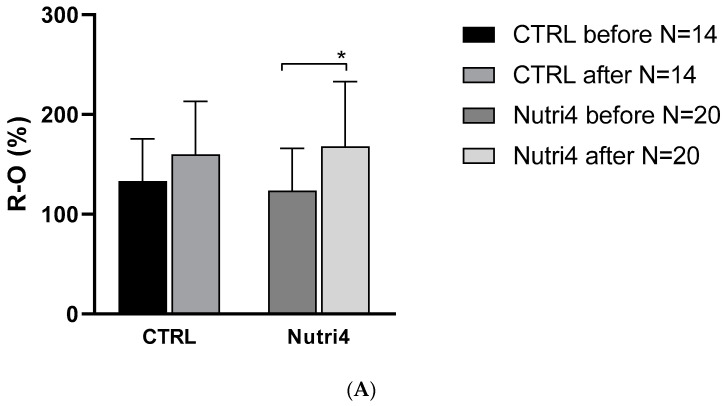

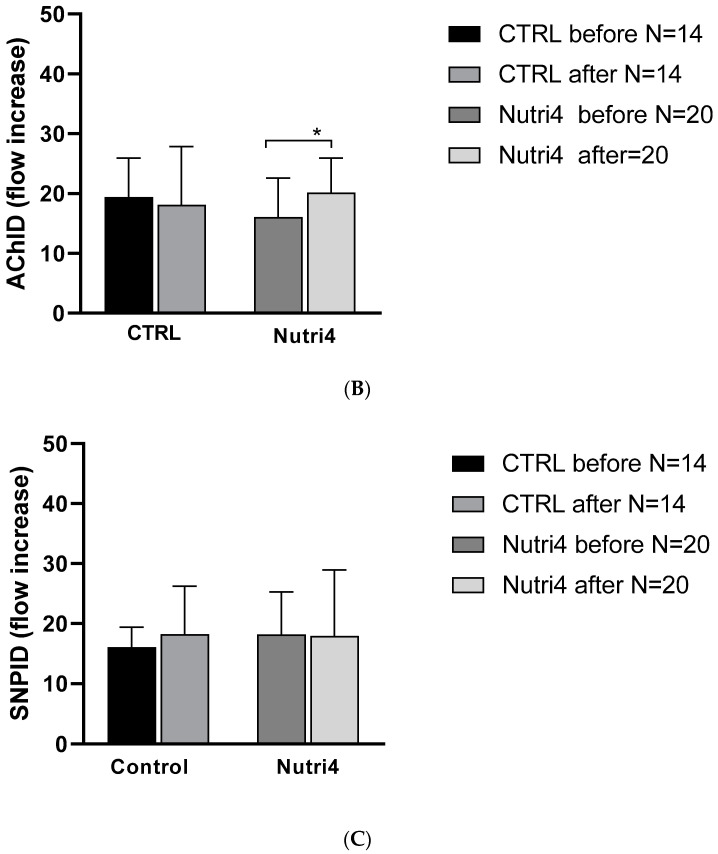
Panel (**A**). Post-occlusive reactive hyperemia (PORH) before and after the dietary protocol. Results are shown as mean ± standard deviation (SD); * *p* < 0.05 before or after within the group (CTRL or Nutri4). Panel (**B**). Forearm skin microcirculation by iontophoresis—AChID before and after the dietary protocol. Results are shown as mean ± standard deviation (SD); * *p* < 0.05 before or after within the group (CTRL or Nutri4). Panel (**C**). Forearm skin microcirculation by iontophoresis sodium nitroprusside-induced dilation—SNPID before and after the dietary protocol.

PORH was significantly increased in the Nutri4 group compared to the CTRL group after the study protocol (Figure 2A). There was no change in microvascular PORH in the CTRL group after the diet protocol compared to the values before the diet (Figure 2A). There was a significant increase in ACh-induced vasodilation in the Nutri4 group after the diet protocol, while consumption of regular eggs did not affect ACh-induced vasodilation in the CTRL group (Figure 2B). Sodium nitroprusside-induced dilation (SNPID) was similar between the groups before and after respective dietary protocols (Figure 2C).

**Figure 3:**
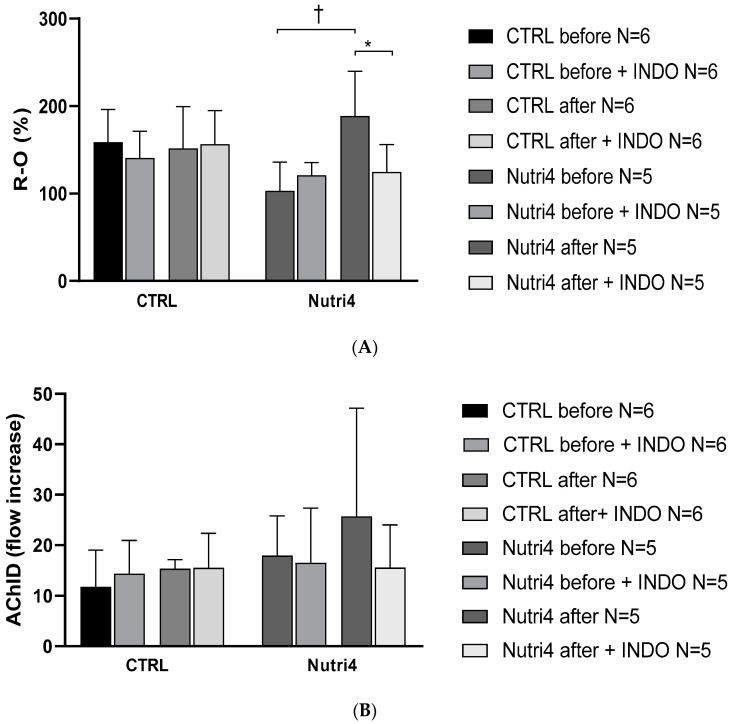
Panel (**A**). Post-occlusive reactive hyperemia (PORH) before and after the dietary protocol with/without administration of indomethacin. This part of the measurements was performed on a separate set of participants, due to measurement design. Results are shown as mean ± standard deviation (SD); * *p* < 0.05 paired *t*-test before and after dietary protocol with the administration of indomethacin. † *p* < 0.05 paired *t*-test before and after dietary protocol without administration of indomethacin. Panel (**B**). Forearm skin microcirculation by iontophoresis-AChID before and after administration of 100 mg indomethacin. This part of the measurement was performed on a separate set of participants, due to the design of the measurement.

PORH, enhanced after Nutri4 eggs consumption was significantly decreased after administration of 100 mg indomethacin in the Nutri4 group, while it remained unchanged in the CTRL group (Figure 3A). AChID remained unchanged in the Nutri4 group and CTRL group after the dietary protocol and after the intake of 100 indomethacin (Figure 3B).

### 3.4. Protein Expression of Enzymes Important in the Mechanisms of Microvascular Reactivity

**Figure 4:**
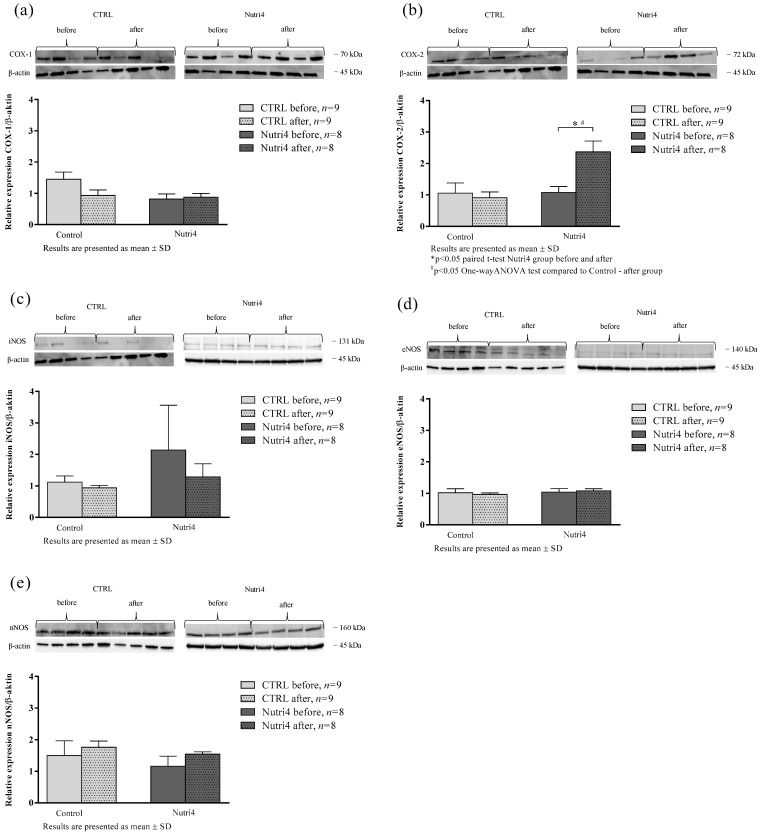
Relative protein expression and representative blots of (**a**) COX-1—cyclooxygenase 1; (**b**) COX-2—cyclooxygenase 2; (**c**) iNOS—inducible nitric oxide synthase; (**d**) eNOS—endothelial nitric oxide synthase; and (**e**) nNOS—neuronal nitric oxide synthase in isolated peripheral blood mononuclear cells (PBMCs), determined by Western blot method. Results are shown as mean ± standard deviation (SD); * *p* < 0.05 paired *t*-test before and after dietary protocol (CTRL or Nutri4); # *p* < 0.05 One-Way ANOVA test difference between the groups.

There was a significant increase in COX-2 protein expression in the Nutri4 group compared to the measurement before dietary protocol and compared to the CTRL group after regular hen egg consumption. There were no significant differences in the protein expressions of nNOS, iNOS, eNOS, and COX-1 in any of the studied groups (Figure 4).

## 4. Discussion

This randomized, double-blind, placebo-controlled interventional study examined for the first time the effects of consumption of functionally enriched hen eggs (enriched with four nutrients; *n*-3 PUFAs, vitamin E, lutein, and Se) on endothelium-dependent and endothelium-independent vascular reactivity in the microcirculation of healthy young subjects. The important findings of the present study are: (1) consumption of Nutri4 hen eggs significantly increased the concentration of *n*-3 PUFAs, significantly decreased the *n*-6/*n*-3 ratio, and increased lutein and vitamin E concentration in the serum of participants, without adversely affecting the lipid profile and other measured biochemical values; and (2) there was a positive effect of consumption of Nutri4 hen eggs on microvascular endothelium-dependent vasodilation, which could be potentially attributed to COX-2 produced metabolites of *n*-3 PUFAs. This is supported by increased expression of COX-2 protein in the Nutri4 group. 

It is well documented that *n*-6 PUFA-derived vasoconstrictive prostaglandins mediate impaired endothelium-dependent responses in conditions such as hypertension and high salt dietary intake [37]. On the other hand, *n*-3 PUFAs compete with *n*-6 PUFAs for pathways for degradation and production of vasoprotective metabolites [37] e.g., they compete for COX-mediated production of prostaglandins and other mediators [38]. In the case of a higher *n*-6/*n*-3 PUFA ratio, prostaglandin series 2 (PGI2), leukotriene series 4 (LTB4), and thromboxane B series 2 (TXB2) are produced, which have vasoconstrictive effects, act as platelet activators and exhibit pro-inflammatory potential [38]. If the ratio goes to higher concentrations of *n*-3 PUFAs, then the production of anti-inflammatory and vasodilator mediators such as prostaglandins series 3 (PGI3), leukotrienes series 5 (LTB5), and thromboxane series 3 (TXA3) dominates [39]. In the present study, there was a significant increase in serum concentration of EPA and DHA, as well as a decrease in the *n*-6/*n*-3 PUFA ratio and increased serum concentration of vitamin E and lutein in the Nutri4 group. Furthermore, DHA significantly increases in CTRL and Nutri4 groups after consumption of respective eggs. However, EPA significantly increased in the Nutri4 group compared CTRL group after the dietary protocol. These results confirmed our previous findings that the consumption of enriched hen eggs provides means to increase nutrients in the blood and potentially alter prostaglandins and leukotrienes balance [29,40]. That had an important impact on microvascular reactivity and microcirculatory blood flow since PORH and AChID were significantly enhanced in the Nutri4 after enriched egg intake, while there was no change in the CTRL. This is in agreement with the study by Stupin et al. where AChID after consumption of *n*-3 PUFA-enriched hen eggs was significantly increased [7]. Hereby, results suggest that mechanisms of enhanced PORH may be related to increased production of COX metabolites, presumably via the COX-2 pathway, since peroral administration of indomethacin, the nonselective blocker of COX-1,2 attenuated PORH, and expression of COX-2 protein increased in Nutri4 group. Interestingly, endothelial selenoproteins control the balance of vascular tone by maintaining the balance of superoxide anion/NO, and the synthesis of eicosanoids by regulating the activity of COX-1, COX-2, and lipoxygenase [39,40]. Previously, we have demonstrated that low concentrations of selenium intake (0.030 mg/kg) can cause increased oxidative stress and decreased AChIR response in rats [41]. Since eggs were enriched with nutrients with anti-oxidant properties (vitamin E, lutein, and selenium), one may speculate that part of the beneficiary effects on microvascular blood flow may be attributed to the alteration of oxidative stress and anti-oxidative defense mechanisms. For example, Barić et al. [8] showed that the use of vitamins E (800 IU/day) and C (1000 mg/day) prevented increased oxidative stress and impairment of microcirculatory function in subjects on an HS diet. However, in the present study, serum markers of oxidative stress were not altered by dietary protocols (data not shown). 

During the three-week dietary protocol, the biochemical, anthropological, and hemodynamic parameters were assessed. Although some of them (e.g., iron, creatinine, and glucose) were significantly changed after dietary protocol, all observed changes were within the reference interval and as such are not physiologically relevant. Similarly, in the CTRL group, there was a significant increase in LDL after a three-week dietary protocol, albeit slightly above referent values. Furthermore, the consumption of eggs has not altered liver enzymes or hsCRP, suggesting a lack of their potential noxious effects.

Coronary heart disease, acute myocardial infarction, and ischemic heart disease are the most often underlined by dyslipidemia. Dietary habits may significantly affect serum lipoproteins [42,43]. It has been shown that in patients with hypertriglyceridemia *n*-3 PUFAs in doses of 2 to 4 g per day lower triglycerides by 25–30%, while total cholesterol does not change [44,45]. In a double-blind controlled study by Tousoulis et al., *n*-3 PUFA supplementation (46% EPA and 34% DHA) of 2 g for 12 weeks improved endothelial function and arterial stiffness in patients with metabolic syndrome [46]. Likewise, long-term consumption of EPA (1600 mg/day) increases NO-dependent and endothelium-independent vasodilation in patients with coronary artery disease [47]. These results are in agreement with our previous study in healthy subjects in which the consumption of *n*-3 PUFA-enriched eggs showed a beneficial effect on microvascular reactivity, endothelium-dependent vasodilatation, and blood pressure, as well as favorable anti-inflammatory properties [6,7]. Thus, the consumption of functional food with the beneficial ratio of *n*-6/*n*-3 PUFAs and with increased content of *n*-3 PUFAs, together with other nutrients may serve as a novel mode in the prevention of cardiovascular diseases. This is supported by the results of the present study that showed significantly increased serum concentration of *n*-3 PUFAs, lutein, and Se and decreased *n*-6/*n*-3 ratio, even in healthy young participants. 

*N*-3 PUFAs have the potential to prevent the development and progression of various diseases, including atherosclerosis and atherosclerosis-related diseases, in part, by improving vascular (endothelial) function [1,2]. Some of these vasculo-protective properties include decreased blood pressure (BP) [2], decreased formation of atherosclerotic lesions [3], increased anti-inflammatory properties [6], and improved endothelium-dependent vasodilation of conduit arteries [7].

Some studies may reduce CV risk in CV patients [9,14,44]. On the other hand, although *n*-3 PUFA functional products are widely available on the market, there is still insufficient data on their possible effects on CV health, and in particular vascular and endothelial function in healthy individuals

There are several limitations to this study. We have not measured the *n*-3 PUFA metabolic products of COX-1 or COX-2 pathways. However, there is a clear increase in protein expression of COX-2 in the Nutri4 group, while expression of other assessed enzymes was not altered with dietary protocol. Therefore, observed enhanced endothelium-dependent vasodilation could be attributed to increased concentration of *n*-3 PUFAs and presumably their metabolites in serum and/or vasculature. Second, although Se levels were not significantly increased, they tended to increase in the Nutri4 group; therefore, the time period of consumption or number of eggs might be extended to observe the benefits of increased Se consumption.

## 5. Conclusions

The present study is the first randomized controlled trial to evaluate the effects of the consumption of functional eggs on microvascular function in healthy humans. Consumption of eggs enriched with *n*-3 PUFAs, vitamin E, selenium, and lutein has a beneficial effect on the microcirculation of healthy young subjects. These beneficial effects can be attributed to mostly *n*-3 PUFA components of enriched eggs. Consumption of enriched eggs enhanced microcirculatory blood flow, which could be, at least partly, mediated by vasodilator metabolites of the COX-2 pathway of *n*-3 PUFA degradation. Likewise, the consumption of hen eggs does not have a negative effect on biochemical parameters, such as serum lipid profile and liver enzyme concentration, thus rendering the eggs a healthy dietary choice.

## Figures and Tables

**Table 1:** Composition of micronutrient ingredients in Regular eggs and Nutri4 eggs.

An L-Grade Egg with an Average Weight of 68 g Has about 60 g of Edible Portion		
Parameters	Regular Eggs	Nutri4 Eggs
Vitamin E (mg)	0.595 ± 0.174	1.098 * ± 0.339
Lutein (mg)	0.11 ± 0.011	0.616 * ± 0.085
Selenium (mg)	0.0183 ± 0.002	0.02305 * ± 0.0012
Fatty acids		
∑SFA	1566 ± 346	1442 ± 185
∑MUFA	1976 ± 189	2419 ± 139
∑*n*-6 PUFA	1263 ± 148	747 ± 46 *
LA	1165 ± 140	702 ± 43
AA	89 ± 9	44 ± 4 *
∑*n*-3 PUFA	146 ± 20	342 ± 25 *
ALA	71 ± 11	189 ± 16 *
EPA	n.d.	19 ± 2 *
DHA	75 ± 11	135 ± 11 *
∑*n*-6/∑*n*-3 PUFA	8.71	2.18 *

Results are shown as mean ± standard deviation (SD). * *p* < 0.05 One-Way ANOVA Regular eggs vs. Nutri4 enriched hen eggs.

**Table 2:** The Effect of Regular and Nutri4 Hen Eggs Consumption on Anthropometric, Hemodynamic and Biochemical Parameters in Healthy Subjects.

Parameter	CTRL		Nutri4	
	Before	After	Before	After
N (W/M)	14 (6/9)		20 (7/13)	
Age (years)	22 ± 3		22 ± 2	
BW (kg)	78 ± 17	77 ± 15	72 ± 15	72 ± 15
BMI (kg/m^2^)	24.3 ± 3.6	24.0 ± 3.2	23.5 ± 3.1	23.5 ± 3.1
WHR	0.84 ± 0.07	0.84 ± 0.07	0.81 ± 0.10	0.81 ± 0.10
SBP (mmHg)	110 ± 17	108 ± 15	109 ± 11	104 ± 14
DBP (mmHg)	72 ± 12	69 ± 4	78 ± 16	72 ± 6
MAP (mmHg)	85 ± 7	82 ± 7	88 ± 13	84 ± 6
HR (beats per minute)	65 ± 8	65 ± 9	78 ± 12 †	78 ± 11 †
Erythrocytes (×10^12^/L)	4.7 ± 0.4	4.8 ± 0.4	4.9 ± 0.5	4.9 ± 0.5
Hemoglobin (g/L)	141 ± 14	141 ± 16	143 ± 12	142 ± 13
Hematocrit (%)	39 ± 3	32 ± 16	40 ± 3	38 ± 10
Leukocytes (×10^9^/L)	6.1 ± 1.9	6.0 ± 1.2	6.3 ± 1.7	5.8 ± 1.9
Thrombocytes (×10^9^/L)	201 ± 65	216 ± 64	233 ± 32	221 ± 32
Urea (mmol/L)	5.0 ± 1.5	5.5 ± 1.3	5.0 ± 1.0	5.7 ± 1.6 *
Creatinine (µmol/L)	86 ± 18	87 ± 14	78 ± 13	75 ± 12
Urate (µmol/L)	330 ± 67	318 ± 88	296 ± 77	289 ± 58
Aspartate aminotransferase (U/L)	25 ± 8	25 ± 6	22 ± 6	22 ± 5
Alanine aminotransferase (U/L)	23 ± 8	22 ± 7	20 ± 15	23 ± 12
Gamma-glutamyltransferase (U/L)	14 ± 4	14 ± 4	17 ± 12	18 ± 11
Sodium (mmol/L)	139 ± 2	139 ± 2	139± 2	139 ± 2
Potassium (mmol/L)	4.2 ± 0.3	4.3 ± 0.3	4.1 ± 0.2	4.2 ± 0.3
Iron (µmol/L)	16.4 ± 6.7	13.8 ± 7.1	21.3 ± 6.3 *	15.5 ± 6.3
Transferrin (g/L)	2.73 ± 0.5	2.78 ± 0.71	2.62 ± 0.34	2.66 ± 0.32
Glucose (mmol/L)	4.6 ± 0.3	4.6 ± 0.5	4.4 ± 0.4	4.9 ± 0.8 *
hsCRP (mg/L)	0.6 ± 0.5	1.1 ± 1.5	1.1 ± 0.9	2.3 ± 0.9
cholesterol (mmol/L)	4.3 ± 0.8	4.6 ± 1.5	4.5 ± 0.5	4.7 ± 0.7
triglycerides (mmol/L)	0.8 ± 0.3	0.9 ± 0.3	1.1 ± 0.5	0.9 ± 0.3
HDL cholesterol (mmol/L)	1.5 ± 0.4	1.4 ± 0.4	1.3 ± 0.2	1.3 ± 0.2
LDL cholesterol (mmol/L)	2.5 ± 0.7	2.9 ± 0.8 *	2.8 ± 0.4	2.9 ± 0.7

Data are presented as mean ± standard deviation (SD). BMI-body mass index; WHR-waist-to-hip ratio; SBP-systolic blood pressure; DBP-diastolic blood pressure; MAP-mean arterial pressure; HR-heart rate; HDL-high-density lipoprotein; LDL-low-density lipoprotein. * *p* < 0.05 before vs. after within the group; † *p* < 0.05 difference between the groups.

**Table 3:** The Effect of Regular and Nutri4 Hen Eggs Consumption on Serum Fatty Acids Profile, Vitamin E, Selenium and Lutein in Healthy Subjects.

Parameter		CTRL		Nutri4	
		Before	After	Before	After
SFA (μmol/L)					
	C4:0 Butyric acid	N/F	N/F	N/F	N/F
	C6:0 Caproic acid	N/F	N/F	N/F	N/F
	C8:0 Caprylic acid	N/F	N/F	N/F	N/F
	C10:0 Capric acid	<LOQ	<LOQ	<LOQ	<LOQ
	C11:0 Undecylic acid	N/F	N/F	N/F	N/F
	C12:0 Lauric acid	<LOQ	20.80	<LOQ	<LOQ
	C13:0 Tridecylic acid	<LOQ	<LOQ	<LOQ	<LOQ
	C14:0 Myristic acid	40.4 ± 9.1	36.3 ± 6.6	50.5 ± 32.2	40.3 ± 11.1
	C15:0 Pentadecylic acid	12.2 ± 1.6	12.7 ± 1.3	13.7 ± 2.4	27.9 ± 39.6
	C16:0 Palmitic Acid	1258 ± 350	1304 ± 332	1295 ± 431	1242 ± 314
	C17:0 Margaric acid	13.5 ± 1.7	13.6 ± 2.6	15.8 ± 3.8	14.7 ± 4.0
	C18:0 Stearic acid	404 ± 111	416 ± 137	515 ± 140	491 ± 132
	C20:0 Arachidic acid	<LOQ	<LOQ	<LOQ	<LOQ
	C21:0 Heneicosanoic acid	N/F	N/F	N/F	N/F
	C22:0 Behenic acid	<LOQ	10.2	<LOQ	<LOQ
	C23:0 Tricosanoic acid	<LOQ	<LOQ	<LOQ	<LOQ
	C24:0 Lignoceric acid	<LOQ	<LOQ	<LOQ	<LOQ
PUFA (μmol/L)					
*n*-5	C14:1[cis-9] Myristoleic acid	<LOQ	<LOQ	11	<LOQ
	C15:1[cis-10] Cis-10-pentadecenoic acid	N/F	N/F	N/F	N/F
*n*-7	C16:1[cis-9] Palmitoleic acid	58.3 ± 14.9	54.1 ± 17.7	95.4 ± 59.3 †	73.6 ± 34.8
	C17:1[cis-10] cis-10-Heptadecenoic acid	N/F	N/F	11.7 ± 3.6	10.7 ± 1.3
*n*-9	C18:1[trans-9] Elaidic acid	N/F	N/F	N/F	N/F
	C18:1[cis-9] Oleic acid	691 ± 218	656 ± 135	872 ± 393	758 ± 279
	C20:1[cis-11] 11-Eicosenoic acid	7.8 ± 2.0	6.2 ± 0.6	8.8 ± 4.4	9.2 ± 2.2
	C22:1[cis-13] Erucic acid	9.2	9.1 ± 0.7	7.4 ± 1.0	7.9
	C24:1[cis-15] Nervonic acid	<LOQ	8.7 ± 0.6	7.2 ± 0.7	7.8 ± 0.7
*n*-6	C18:2[trans-9,12] Linoelaidic acid	N/F	N/F	12,5	N/F
	C18:2[cis-9,12] Linoleic acid	1148 ± 300	1170 ± 298	1223 ± 290	1277 ± 461
	C18:3[cis-6,9,12] gamma-Linolenic acid	19.9 ± 6.7	17.2 ± 3.7	27.3 ± 13.8	23.2 ± 7.4
	C21:2[cis-11,14] Eicosadienoic acid	8.6 ± 1.9	9.5 ± 2.6	12.3 ± 5.8	11.1 ± 3.6
	C20:3[cis-8,11,14] Dihomo-gamma-linolenic acid	54.1 ± 16.2	54.1 ± 12.9	78.7 ± 42.6	69.3 ± 42.0
	C20:4[cis-5,8,11,14] Arachidonic acid	298 ± 52	354 ± 53	390 ± 127	369 ± 106
	C22:2[cis-13,16] 13,16-Docosadienoic acid	N/F	N/F	N/F	N/F
*n*-3	C18:3[cis-9,12,15] alpha-Linolenic acid	17.2 ± 7.7	16.0 ± 5.1	16.4 ± 6.3	21.1 ± 8.0
	C20:3[cis-11,14,17] 11,14,17-Eicosatrienoic acid	N/F	N/F	N/F	N/F
	C20:4[cis-5,8,11,14] Eicosa-5,8,11,14,17-pentaenoic acid	15.1 ± 6.9	14.1 ± 4.6	13.7 ± 2.9	18.6 ± 5.6 *†
	C22:6[cis-4,7,10,13,16,19] cis-4,7,10,13,16,19-Docosahexaenoic acid	54.0 ± 19.9	89.9 ± 37.7 *	65.6 ± 25.1	132.1 ± 64.7 *
*n*-6/*n*-3 ratio		11.1	8.3	10.5	6.3 *
Vitamin E μg/mL		10.27 ± 3.67	10.30 ± 3.687	6.63 ± 3.22	11.26 ± 2.87 *
Selenium μg/L		62.44 ± 9.37	66.37± 10.09	64.88 ± 17.65	68.88 ± 3.082
Lutein μmol/L		0.199 ± 0.104	0.199 ± 0.202	0.153 ± 0.073	0.232 ± 0.078 *

Results are expressed as mean ± standard deviation (SD). <LOQ—below limit of quantification; N/F—not found. * *p* < 0.05 before vs. after within the group (CTRL or Nutri4); † *p* < 0.05 difference between the groups.

## Data Availability

The data presented in this study are available on request from the corresponding author.

## References

[1] Auger C., Said A., Nguyen P.N., Chabert P., Idris-Khodja N., Schini-Kerth V.B. (2016). Potential of food and natural products to promote endothelial and vascular health. J. Cardiovasc. Pharmacol..

[2] Ulu A., Lee K.S.S., Miyabe C.B., Yang J., Hammock B.G., Dong H. (2014). An omega-3 epoxide of docosahexaenoic acid lowers blood pressure in angiotensin-II—Dependent hypertension. J. Cardiovasc. Pharmacol..

[3] Renier G., Skamene E., DeSanctis J., Radzioch D. (1993). Dietary n-3 polyunsaturated fatty acids prevent the development of atherosclerotic lesions in mice. Modulation of macrophage secretory activities. Arter. Thromb. A J. Vasc. Biol..

[4] Kris-Etherton P.M., Harris W.S., Appel L.J. (2002). Fish consumption, fish oil, omega-3 fatty acids, and cardiovascular disease. Circulation.

[5] Schachinger V., Britten M.B., Zeiher A.M. (2000). Prognostic impact of coronary vasodilator dysfunction on adverse long-term outcome of coronary heart disease. Circulation.

[6] Stupin A., Mihalj M., Kolobarić N., Šušnjara P., Kolar L., Mihaljević Z., Matić A., Stupin M., Jukić I., Kralik Z. (2020). Anti-inflammatory potential of n-3 polyunsaturated fatty acids enriched hen eggs consumption in improving microvascular endothelial function of healthy individuals—Clinical trial. Int. J. Mol. Sci..

[7] Stupin A., Rasic L., Matic A., Stupin M., Kralik Z., Kralik G., Grcevic M., Drenjancevic I. (2018). Omega-3 polyunsaturated fatty acids-enriched hen eggs consumption enhances microvascular reactivity in young healthy individuals. Appl. Physiol. Nutr. Metab..

[8] Barić L., Drenjančević I., Mihalj M. (2020). Enhanced antioxidative defense by vitamins C and E consumption prevents 7-day high-salt diet-induced microvascular endothelial function impairment in young healthy individuals. J. Clin. Med..

[9] Ćuric Ž.B., Masle A.M., Kibel A. (2021). Effects of n-3 polyunsaturated fatty acid-enriched hen egg consumption on the inflammatory biomarkers and microvascular function in patients with acute and chronic coronary syndrome—A randomized study. Biology.

[10] Armah C.K., Jackson K.G., Doman I., James L., Cheghani F., Minihane A.M. (2008). Fish oil fatty acids improve postprandial vascular reactivity in healthy men. Clin. Sci..

[11] Jiang Q. (2014). Natural forms of vitamin E: Metabolism, antioxidant and anti-inflammatory activities and the role in disease pre-vention and therapy. Free Radic Biol. Med..

[12] Yan J.-H., Guan B.-J., Gao H.-Y., Peng X.-E. (2018). Omega-3 polyunsaturated fatty acid supplementation and non-alcoholic fatty liver disease. Medicine.

[13] Traber M.G., Stevens J.F. (2011). Vitamins C and E: Beneficial effects from a mechanistic perspective. Free. Radic. Biol. Med..

[14] Leme Goto P., Cinato M., Merachli F. (2020). In vitro and in vivo cardioprotective and metabolic efficacy of vitamin E TPGS/Apelin. J. Mol. Cell. Cardiol..

[15] Mehrdad R., Reza J.N.M., Tourandokht B. (2013). Endothelium-dependent effect of sesame seed feeding on vascular reactivity of streptozotocin-diabetic rats: Underlying mechanisms. Iran. J. Pharm. Res..

[16] Kipp A.P., Strohm D., Brigelius-Flohé R. (2015). Revised reference values for selenium intake. J. Trace Elem. Med. Biol..

[17] Zhao J., Xing H., Liu C., Zhang Z., Xu S. (2015). Effect of selenium deficiency on nitric oxide and heat shock proteins in chicken erythrocytes. Biol. Trace Element Res..

[18] Kuruppu D., Hendrie H.C., Yang L., Gao S. (2013). Selenium levels and hypertension: A systematic review of the literature. Public Health Nutr..

[19] Rees K., Hartley L., Day C. (2013). Selenium supplementation for the primary prevention of cardiovascular disease. Cochrane Database Syst. Rev..

[20] Hajizadeh-Sharafabad F., Ghoreishi Z., Maleki V., Tarighat-Esfanjani A. (2019). Mechanistic insights into the effect of lutein on atherosclerosis, vascular dysfunction, and related risk factors: A systematic review of in vivo, ex vivo and in vitro studies. Pharmacol. Res..

[21] Ahn Y.J., Kim H. (2021). Lutein as a modulator of oxidative stress-mediated inflammatory diseases. Antioxidants.

[22] Drenjancevic I. Effect of Enriched QUARTET® Hen Eggs on Cardiovascular Function in Cardiovascular Patients and Healthy Individuals. Clinical Trial Registration study/NCT04564690, clinicaltrials.gov. NCT04564690.

[23] Suresh K. (2011). An overview of randomization techniques: An unbiased assessment of outcome in clinical research. J. Hum. Reprod. Sci..

[24] Kralik Z., Kralik G., Grčević M., Kralik I., Gantner V. (2018). Physical-chemical characteristics of designer and conventional eggs. Braz J. Poult. Sci..

[25] Kralik Z., Kralik G., Galović O. (2011). Nutricines content in table eggs of Croatian producers. Book of Abstracts of the 13th International Scientific and Professional Conference with Food to Health = Knjiga Sažetaka s 13 Međunarodnog Znanstveno-Stručnog skupa Hranom do Zdravlja. https://www.bib.irb.hr/1147732.

[26] Cavka A., Cosic A., Jukic I., Jelakovic B., Lombard J.H., Phillips S.A., Seric V., Mihaljevic I., Drenjancevic I. (2015). The role of cyclo-oxygenase-1 in high-salt diet-induced microvascular dysfunction in humans. J. Physiol..

[27] Stupin M., Stupin A., Rasic L., Cosic A., Kolar L., Seric V., Lenasi H., Izakovic K., Drenjancevic I. (2017). Acute exhaustive rowing exercise reduces skin microvascular dilator function in young adult rowing athletes. Eur. J. Appl. Physiol..

[28] Walser B., Giordano R.M., Stebbins C.L. (2006). Supplementation with omega-3 polyunsaturated fatty acids augments brachial artery dilation and blood flow during forearm contraction. Eur. J. Appl. Physiol..

[29] Kolobarić N., Drenjančević I., Matić A. (2021). Dietary intake of n-3 PUFA-enriched hen eggs changes inflammatory markers’ concentration and Treg/Th17 cells distribution in blood of young healthy adults—A randomised study. Nutrients.

[30] Mihaljević Z., Matić A., Stupin A., Frkanec R., Tavčar B., Kelava V., Bujak I.T., Kolobarić N., Kibel A., Drenjančević I. (2020). Arachidonic acid metabolites of CYP450 enzymes and HIF-1α modulate endothelium-dependent vasorelaxation in sprague-dawley rats under acute and intermittent hyperbaric oxygenation. Int. J. Mol. Sci..

[31] Matic A., Jukic I., Stupin A., Baric L., Mihaljevic Z., Unfirer S., Bujak I.T., Mihaljevic B., Lombard J.H., Drenjancevic I. (2018). High salt intake shifts the mechanisms of flow-induced dilation in the middle cerebral arteries of Sprague-Dawley rats. Am. J. Physiol. Circ. Physiol..

[32] Mihaljević Z., Matić A., Stupin A., Rašić L., Jukić I., Drenjančević I. (2018). Acute hyperbaric oxygenation, contrary to intermittent hyperbaric oxygenation, adversely affects vasorelaxation in healthy sprague-dawley rats due to increased oxidative stress. Oxidative Med. Cell Longev..

[33] Wang L.Y., Summerhill K., Rodriguez-Canas C., Mather I., Patel P., Eiden M., Young S., Forouhi N.G., Koulman A. (2013). Development and validation of a robust automated analysis of plasma phospholipid fatty acids for metabolic phenotyping of large epidemiological studies. Genome Med..

[34] Jargar J.G., Dhundasi S.A., Punekar M.D. (2017). Status of α-tocopherol concentration and oxidative stress in infertile females of Vija-yapur District, northern Karnataka. Natl. J. Physiol. Pharm. Pharmacol..

[35] Tzeng M.-S., Yang F.-L., Wang-Hsu G.-S., Chen B.-H. (2004). Determination of major carotenoids in human serum by liquid chromatography. J. Food Drug Anal..

[36] Leeson S., Caston L. (2004). Enrichment of eggs with lutein. Poult. Sci..

[37] Das U.N. (2018). Arachidonic acid in health and disease with focus on hypertension and diabetes mellitus: A review. J. Adv. Res..

[38] Schmitz G., Ecker J. (2008). The opposing effects of n-3 and n-6 fatty acids. Prog. Lipid Res..

[39] Arnold C., Konkel A., Fischer R. (2010). Cytochrome P450-dependent metabolism of w-6 and w-3 long-chain polyunsaturated fatty acids. Pharmacol. Rep..

[40] Kolar L., Stupin M., Stupin A. (2021). Does the endothelium of competitive athletes benefit from consumption of n-3 poly-unsaturated fatty acid-enriched hen eggs?. Prev. Nutr. Food Sci..

[41] Stupin A., Cosic A., Novak S., Vesel M., Jukic I., Popovic B., Karalic K., Loncaric Z., Drenjancevic I. (2017). Reduced dietary selenium impairs vascular function by increasing oxidative stress in Sprague-Dawley rat aortas. Int. J. Environ. Res. Public Health.

[42] Minieri M., Di Nardo P. (2007). Nutrients: The environmental regulation of cardiovascular gene expression. Genes Nutr..

[43] Iacoviello L., Santimone I., Latella M.C., de Gaetano G., Donati M.B. (2008). Nutrigenomics: A case for the common soil between cardiovascular disease and cancer. Genes Nutr..

[44] Marchioli R., Barzi F., Bomba E. (2002). Early protection against sudden death by n-3 polyunsaturated fatty acids after myocardial infarction. Circulation.

[45] Streppel M.T., Ocké M.C., Boshuizen H.C., Kok F.J., Kromhout D. (2008). Long-term fish consumption and n-3 fatty acid intake in relation to (sudden) coronary heart disease death: The Zutphen study. Eur. Heart J..

[46] Tousoulis D., Plastiras A., Siasos G., Oikonomou E., Verveniotis A., Kokkou E., Maniatis K., Gouliopoulos N., Miliou A., Paraskevopoulos T. (2014). Omega-3 PUFAs improved endothelial function and arterial stiffness with a parallel antiinflammatory effect in adults with metabolic syndrome. Atherosclerosis.

[47] Tagawa H., Shimokawa H., Tagawa T. (1999). Long-term treatment with Eicosapentaenoic acid augments both nitric oxide-mediated and non-nitric oxide-mediated endothelium-dependent forearm vasodilatation in patients with coronary artery disease. J. Cardiovasc. Pharmacol..

